# Influence of Oil Contamination on Physical and Biological Properties of Forest Soil After Chainsaw Use

**DOI:** 10.1007/s11270-015-2649-2

**Published:** 2015-10-31

**Authors:** Anna Klamerus-Iwan, Ewa Błońska, Jarosław Lasota, Agnieszka Kalandyk, Piotr Waligórski

**Affiliations:** Faculty of Forestry, Department of Forest Engineering, University of Agriculture in Kraków, Al. 29 Listopada 46, 31-425 Kraków, Poland; Faculty of Forestry, Department of Forest Soil, University of Agriculture in Krakow, Al. 29 Listopada 46, 31-425 Kraków, Poland; The F. Górski Institute of Plant Physiology, Polish Academy of Sciences, Kraków, ul. Niezapominajek 21, 30-233 Kraków, Poland

**Keywords:** Polycyclic aromatic hydrocarbons (PAHs), Enzyme activity, Earthworms, Mechanization in forestry, Principal component analysis (PCA)

## Abstract

Forestry works using chainsaws result in up to 7 million liters of various mineral oils being soaked annually into forest soils. These substances, containing a complex mixture of polycyclic aromatic hydrocarbons (PAHs), are highly toxic. The aim of the study was to determine the effect of oil contamination with PAHs on the physical and biological properties of forest soils. The study area was located in southern Poland in the Miechów forest district. The experiment was conducted on four treatment blocks with various amounts of oil addition. The study included the determination of PAH content, dehydrogenase and urease activity, and biomass of earthworms. Physical properties were determined using the dryer method and Kopecky rings of 250 cm^3^ volume. The results obtained confirmed the hypothesis that oil contamination with PAHs modified the physical properties of forest soils and oil had a negative impact on enzyme activity in soil. Enzyme activity in the studied soils was negatively correlated with PAH content. Earthworm population density reflected the contamination level of oil-contaminated soils.

## Introduction

Soil contamination by oil from chainsaws is a serious problem in forest management. Most jobs performed in forests require the use of chainsaws. It is estimated that with logging in Poland yielding around 30 million m^3^ of wood per year, approximately 6 million dm^3^ of oil—both original, first-time use oil, and re-used oil after reprocessing—penetrates into the environment. Environmental contamination by oil is especially dangerous in the case of used oil because of its strong toxic properties (Giefing 1991; Rudko and Rybczyński 2010). Mechanization in forestry leads to the emission of pollutants into the natural environment, including polycyclic aromatic hydrocarbons (PAHs). Oils are the source of PAHs and the PAHs contained in oil penetrate into the soil environment (Lipińska et al. 2013). The soil system is considered the most important long-term repository for PAHs, and it is also considered to be a steady indicator of the state of environmental pollution (Nam et al. 2003; Mueller and Shann 2006). PAHs affect the activity of soil enzymes, which can be used to evaluate soil microbial properties (Shen et al. 2006). The activity of soil enzymes is one of the approved parameters used for the evaluation of soil quality polluted with organic compounds, including PAHs (Lipińska et al. 2014). The activity of urease appears to be more sensitive to pollution than other soil enzymes (Bååth 1989). Dehydrogenase activity is the most frequently used test for determining the influence of various pollutants on the microbiological quality of soil (Sannino and Gianfreda 2001; Baran et al. 2004).

Contamination with hydrocarbons can have a profound effect on soil fauna (Dendooven et al. 2011). Several authors reported the negative effect of PAHs on the survival and reproduction of earthworms (Brown et al. 2004; Contreras-Ramos et al. 2006).

Oil pollution might affect soil physical properties. Pore spaces might be clogged, which could reduce soil aeration and water infiltration and increase bulk density, subsequently affecting plant growth. Oils that are denser than water might reduce and restrict soil permeability (Abosede 2013).

Beech (*Fagus sylvatica*) is a forest species of high economic importance. In recent years, this species has frequently been introduced into Polish forests. After harvesting of trees, foresters plant new seedlings and they want to know the short-term negative effects of the mineral oil from chainsaw use on soil properties. The growth and quality of seedlings depends on the soil properties.

The aim of the study was to determine the effect of oil contamination with PAHs on the physical and biological properties of forest soils characterized by the same pH, clay, and carbon contents. The study involved the evaluation of the influence of different amounts of oil on soil moisture and aeration conditions, enzyme activity, and density of earthworms. We hypothesized that: (1) contamination of soil with PAHs would have a negative impact on dehydrogenase and urease activity of forest soil; (2) earthworm population density would reflect the state of oil-contaminated soils; (3) contamination by oil containing PAHs would modify the moisture and aeration characteristics of forest soils.

## Materials and Methods

### Soil Sampling Sites

Sample plots were located in southern Poland in the vicinity of Kraków, within the Miechów forest district (50.3000°N, 19.9833°E). The study area is characterized by the following climate conditions: the average precipitation in the months of July and August is 87.5 mm and the average temperature is 16.6 °C. Sample plots were located in an area with a predominance of highland loess of aeolian origin. The test area was dominated by Cambisols (WRB 2002). Table 1 shows the soil properties for the test site. A detailed description of the soil profile was carried out; samples were taken from each genetic horizon to characterize the basic soil properties (soil pH in H_2_O and 1 M KCl solutions, hydrolytic acidity, exchangeable acidity, exchangeable Al, total nitrogen (N) and carbon (C) contents and calculated C/N ratio, granulometric composition).

**Table 1:** Properties of investigated soil

Depth	*C*	*N*	C/N	pH H_2_O	pH KCl	*Y*	*H*_w_	Al	*S*	*V*	Sand	Dust	Clay	Humus type	TSL
0–15	4.81	0.54	9	4.82	4.12	8.12	0.83	0.41	6.7	35	5	88	7	Mull	Lwyżśw
15–45	0.54	0.02	27	4.61	3.74	4.45	4.31	4.22	1.1	18	7	86	7		
45–100	n.d.	n.d.	n.d.	4.91	3.74	4.89	4.21	4.18	7.5	58	7	83	10		
100–120	n.d.	n.d.	n.d.	5.23	3.98	2.21	0.84	0.63	8.8	80	7	84	9		

C total organic carbon (%), N total nitrogen (%), C/N C/N ratio, S sum of exchangeable base cations (cmol(+) kg^−1^), V saturation of base cations (%), Y hydrolytic acidity (cmol(+) kg^−1^), Hw exchangeable acidity (cmol(+) kg^−1^), Al exchangeable aluminum (mg 100 g^−1^)

*TSL* type of forest site, Lwyżśw upland broadleaf forest site, *n.d*. no determined

Beech (*F. sylvatica*) was the dominant species in the study area stands. The study area was divided into four blocks (I, II, III, and IV) of 2 × 2 m (4 m^2^). Oil (50 g/m^2^ (D1)) was administered to the first block, 100 g/m^2^ (D2) to the second block, and 200 g/m^2^ (D3) to the third block. Block IV served as a control (*C*). The experiment was carried out during the vegetation period, in the months of July and August 2014. Various amounts of oil were added to the soil in July, and samples for laboratory analysis were collected a month later. Oil was applied to the soil using a sprinkler.

### Physical Analyses

Soil moisture (Wv expressed as % of volume, or Ww expressed as % of weight), capillary water capacity (CWCw expressed as % of weight, or CWCv expressed as % of volume), and bulk density (Bd) were determined using the dryer method with Kopecky rings of 250 cm^3^ volume (Ostrowska et al. 1991). Density of soil solid phase was determined by pycnometry, while total porosity was calculated on the basis of density of the solid phase and soil density. Air-filled porosity was calculated based on the total porosity and capillary water capacity.

### Biological Analyses

Dehydrogenase (EC 1.1.1.1) and urease (EC 3.5.1.5) activities were determined. Five replicate soil samples were taken from each block at a depth of 0–10 cm for a total of 20 samples. Dehydrogenase activity was detected using Lenhard’s method according to the Casida procedure (Alef and Nannipieri 1995); enzyme activity was expressed as mg triphenyl formazan (TPF) formed per 100 g soil per 24 h. Urease activity was determined using the method of Tabatabai and Bremner (1972; as cited in Alef and Nannipieri 1995); enzyme activity was expressed as μg N-NH_4_ formed per 1 g of soil per 2 h.

To determine earthworm density, two methods were used: manual sorting and the formalin extraction method (Clapperton et al. 2007). Five replicates were used for the calculation of earthworm density and biomass for each treatment.

### Determination of PAHs

The soil was air dried at 40 °C for 48 h and then ground in a mortar. Approximately 5 g was weighed from each sample, and then PAHs were extracted using 35 mL of *n*-hexane. The samples were sonicated and centrifuged (4000*g*, 5 min), the supernatant was collected, and the extraction was repeated with the application of 20 mL of *n*-hexane. Both supernatants were combined and evaporated to dryness using a rotary evaporator. The residue was dissolved in acetonitrile and analyzed for PAHs by high-performance liquid chromatography (HPLC). The Agilent Technologies 1260 system was equipped with a fluorescence detector and Agilent Technologies Eclipse XDB-C18 5 μm, 4.6 × 150 mm HPLC column. The mobile phases were water (A) and acetonitrile (B) at a flow rate of 1 mL/min. The sample injection volume was 5 μL. Compounds were eluted using the following gradient: 0–5 min 20:80 A:B→13:87 A:B, 5–7.5 min held at 13:87 A:B, 7.5–15 min 13:87 A:B→0:100 A:B. Ten-point calibration curves were prepared for the following compounds (retention time and excitation and emission wavelengths are provided in brackets): phenanthrene (4.77 min, 252 nm, 365 nm), pyrene (6 min, 240 nm, 390 nm), chrysene (7 min, 270 nm, 380 nm), benzo[a]pyrene (10 min, 265 nm, 410 nm) and dibenz[a,h]anthracene (11 min, 295 nm, 405 nm). These compounds were chosen because of their high content in raw oil and petrochemical products. We also analyzed the PAHs in oil. One hundred microliters of oil was extracted with 10 mL acetonitrile, vortexed and sonicated for 20 min. The sample was then centrifuged (20,000*g* for 20 min), and the supernatant was analyzed with the same HPLC method as described above.

### Statistical Analyses

To reduce the number of variables in the statistical data set and to visualize the multivariate data set as a set of coordinates in a high-dimensional data space, principal component analysis (PCA) was used. The PCA method was also used to interpret other factors, depending on the type of data set. In PCA analysis, the physical properties and enzyme activities of soil were used. Differences between the mean values in soil groups were evaluated with the Tukey’s test (*P* < 0.05). All statistical analyses were performed with Statistica 10 software (2010).

## Results

### Soil Physical Properties

Changes in selected properties of soil resulting from the addition of chainsaw oil to the soil were observed. Slightly lower moisture content was noted in the soil samples collected from the block where the highest amount of oil was added. However, the relative moisture expressed in relation to the dry sample weight was not significantly different between treatments (Table 2). Capillary water content increased slightly in correlation with the amount of oil added to the soil (Table 2). Soil bulk density increased with increased soil contamination with chainsaw oil and total porosity and air-filled porosity simultaneously decreased. Oil at 100 g/m^2^ caused a decrease of 4 % in air-filled porosity, and the highest amount of oil (200 g/m^2^) resulted in a decrease in air-filled porosity of nearly 10 % compared with unpolluted soil (Table 2).

**Table 2:** Physical properties of soil with different dose of oil

Dose of oil	Wv	Ww	Pwk	Pwv	Bd	Pt	Pa
D1	10.42^a^ ± 1.39	7.0^a^ ± 1.03	18.8^a^ ± 1.91	27.8^a^ ± 2.46	1.48^a^ ± 0.02	39.5^b^ ± 0.91	11.7^bc^ ± 1.67
D2	10.94^a^ ± 0.73	7.0^a^ ± 0.62	18.4^a^ ± 1.12	28.6^a^ ± 1.22	1.51^ab^ ± 0.09	38.4^ab^ ± 3.60	9.7^b^ ± 2.86
D3	9.74^a^ ± 0.97	6.5^a^ ± 0.76	19.7^a^ ± 1.97	29.7^a^ ± 1.50	1.59^b^ ± 0.04	35.2^a^ ± 1.70	5.5^a^ ± 1.89
C	11.26^a^ ± 2.47	6.9^a^ ± 1.22	17.3^a^ ± 1.26	27.9^a^ ± 1.53	1.42^a^ ± 0.08	41.9^b^ ± 0.80	14.0^c^ ± 1.41

Different small letters in the upper index of the mean values mean significant differences

Dose of oil: D1 50 g/m^2^, D2 100 g/m^2^, D3 200 g/m^2^, C 0 (control); Wv relative moisture content in volume percent, Ww relative moisture content in weight percent, Pwk capillary water capacity in weight percent, Pwv capillary water capacity in volume percent, Bd bulk density of soil (g cm^−3^), Pt total porosity of soil in volume percent, Pa non-capillary porosity in volume percent

Water resistance of soil aggregates is another property of soil that can be modified by the presence of oily substances containing aromatic hydrocarbons. The addition of oil to soil resulted in the increased water resistance of different size aggregates; this influence was stronger the higher the amount of oil introduced into the soil (Table 3). Oil at 200 g/m^2^ resulted in almost complete resistance of aggregates to disintegration in water, even after 24 h immersion in water. In contrast, small and medium-sized aggregates from soil not treated with oil disintegrated by 40–60 % after 1 h immersion and after 24 h immersion, no permanent aggregates were noted (Table 3).

**Table 3:** Water resistant of aggregate in soil with different of dose oil

Measurement time (h)	Dose of oil	Diameter of soil aggregates (mm)			
		>10	5–10	3–5	2–3
1	D1	75	80	80	80
	D2	75	60	60	80
	D3	100	100	100	100
	C	75	60	40	60
5	D1	75	80	60	60
	D2	75	40	40	60
	D3	100	100	100	100
	C	75	40	20	40
24	D1	75	60	0	40
	D2	75	40	20	0
	D3	100	100	75	100
	C	50	20	0	0

Dose of oil: D1 50 g/m^2^, D2 100 g/m^2^, D3 200 g/m^2^, C 0 (control)

### Soil Biological Properties

Oil contamination of soil had significant effects on soil biological activity. A strong reduction in dehydrogenase activity was observed at 100 g/m^2^, and at 200 g/m^2^, dehydrogenase activity was reduced by approximately 50 % compared with the activity in the control soil (Table 4). A similar pattern was observed for urease activity. The lowest amount of oil (50 g/m^2^) resulted in a slight decrease in urease activity, while activity was reduced by 40 and 50 % at 100 and 200 g/m^2^, respectively, compared with the control. The chainsaw oil used in the experiment had a major influence on the density and biomass of earthworms. Even the smallest amount of oil (50 g/m^2^) caused a decline in the population of earthworms from >40 specimens/m^2^ in the control block to ∼9 specimens/m^2^. Oil at 100 g/m^2^ affected the species composition of earthworms; this was confirmed by lower biomass of these animals (with similar numbers, the biomass decreased from >3.0 to 0.4 g/m^2^; Table 4).

**Table 4:** Enzyme activity and quantity of earthworms in the soil with different dose of oil

Dose of oil	DH	UR	DE	BE
D1	25.96^c^ ±1.50	0.20^c^ ±0.02	8.67^c^ ±0.58	3.37^b^ ±0.00
D2	19.11^b^ ±1.70	0.15^b^ ±0.01	7.33^b^ ±0.58	0.41^a^ ±0.06
D3	15.93^a^ ±3.01	0.12^a^ ±0.01	0.00^a^ ±0.00	0.00^a^ ±0.00
C	27.80^c^ ±4.68	0.25^d^ ±0.00	43.33^d^ ±0.58	12.08^c^ ±0.86

Different small letters in the upper index of the mean values mean significant differences

Dose of oil: D1 50 g m^−2^, D2 100 g m^−2^, D3 200 g m^−2^, C 0 (control), *DH* dehydrogenase activity (μM TPF kg^−1^ soil h^−1^), *UR* urease activity (mM N-NH_4_ kg^−1^ soil h^−1^), *DE* density earthworms (the number of pieces m^−2^), *BE* biomass of earthworms (g m^−2^)

### Soil PAH Content

The contents of selected PAHs in oil and soil on the first day of the experiment were presented in Table 5. The PAH contents in oil-contaminated soils after 1 month were presented in Table 6. Among the tested soils, the highest hydrocarbon contents were recorded for chrysene and pyrene. Hydrocarbon contents were higher in oil-contaminated soils than in the control soil. The highest concentrations of chrysene (0.1756 μg/g), phenanthrene, pyrene, and benzo(a)pyrene were observed in soils with highest amount of oil (D3). The concentration of phenanthrene in soil after 1 month was the most reduced compared with the starting concentration, indicating substantial degradation of this compound during the incubation period. Pyrene, chrysene, and benzo(a)pyrene were strongly absorbed to soil and thus poorly bioavailable for degradation (Tables 5 and 6).

**Table 5:** Selected polycyclic aromatic hydrocarbons in oil (μg ml) and soil with different dose of oil in first day experiment (μg g of soil)

	Phenanthrene	Pyrene	Chryzene	Benzo-a-pyrene	Dibenzo-a,h-anthracene
D1	0.0547	0.0413	0.1129	0.0042	0.000
D2	0.0785	0.0527	0.1326	0.0053	0.000
D3	0.1262	0.0756	0.1721	0.0075	0.000
PAHs in oil	6.1000	2.9301	5.0500	0.2800	0.000

Dose of oil: D1 50 g/m^2^, D2 100 g/m^2^, D3 200 g/m^2^, C 0 (control)

**Table 6:** Selected polycyclic aromatic hydrocarbons in soil with different dose of oil after 1 month

Dose of oil	Phenanthrene	Pyrene	Chryzene	Benzo-a-pyrene	Dibenzo-a,h-anthracene
D1	0.0356	0.0393	0.1157	0.0040	0.0088
D2	0.0337	0.0500	0.1275	0.0020	0.0082
D3	0.0414	0.0622	0.1756	0.0062	0.0156
C	0.0308	0.0298	0.0931	0.0031	0.0056

Dose of oil: D1 50 g/m^2^, D2 100 g/m^2^, D3 200 g/m^2^, C 0 (control)

### PCA Analysis

**Fig. 1:**
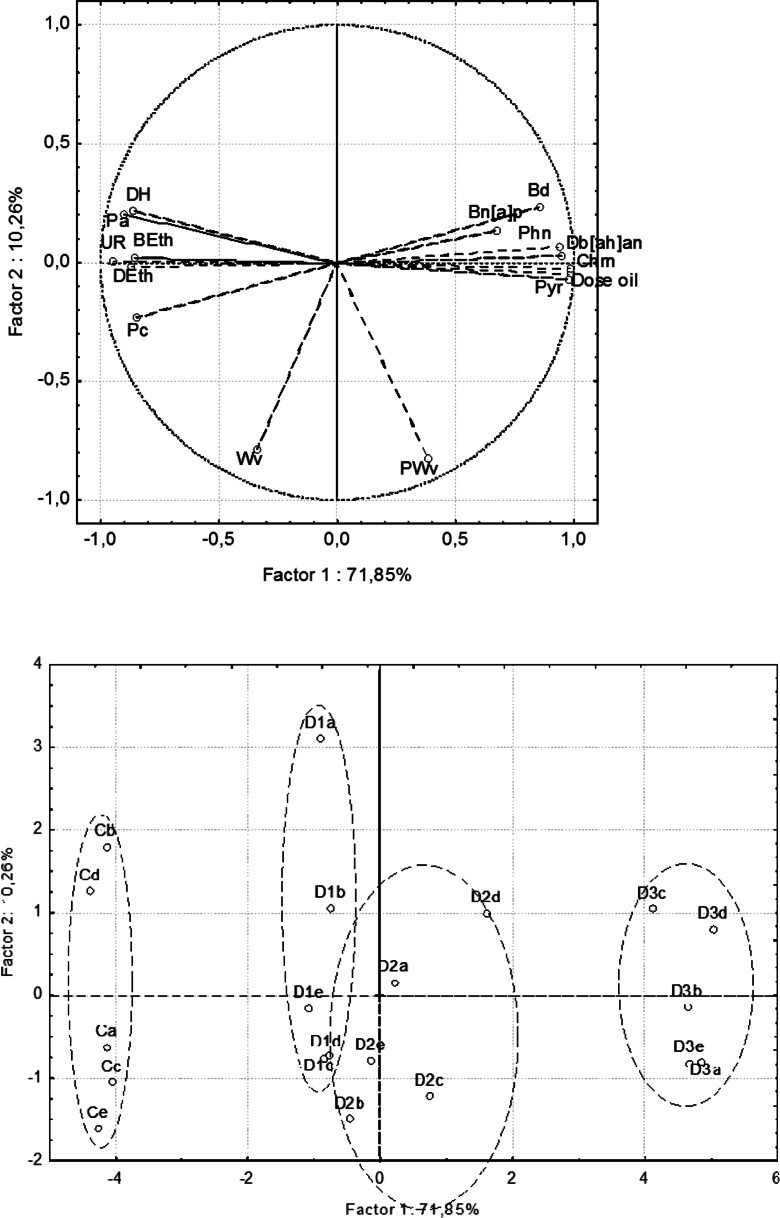
Factorial plan and projection of variables in the soil properties on the factor-plane 1 × 2

Two main factors were selected that explained 82.11 % of the total variance of the variables (Fig. 1). Factor 1, which represented 71.85 % of the variation, was strongly correlated with enzyme activity, biomass and density of earthworms, and PAH content. Factor 2, which explained 10.26 % of the variation, was correlated with the soil water content and capillary water capacity.

Biological properties of the studied soils were negatively correlated with PAH content. Enzyme activity and biomass and density of earthworms were positively correlated with porosity (Fig. 1).

## Discussion

During this study, changes in soil physical properties because of the application of oil were observed. Slightly lower moisture content (expressed as soil volume %) was recorded in the soil samples collected from the block where the highest amount of nebulized oil was applied. Capillary water capacity slightly increased with the amount of oil introduced into the soil. It is difficult to say whether this trend was directly related to the change in physical properties of soil when oil was introduced into it, or whether the differences were within the acceptable range of variation for a single stand of trees under natural conditions. The increase in capillary water capacity correlated with the increasing amount of oil added to the soil could be explained by a decrease in the diameter of larger capillaries, and consequently stronger water-holding capacity of these capillaries, or inhibition of their drainage capacity. Khamehchiyan et al. (2007) and Kuyukina et al. (2005) reported that high concentrations of crude oil in soil could clog soil pores and reduce water and oxygen penetration. According to Abosede (2013), crude oil might have negative effects on some soil physical properties including decreased pore spaces, saturated hydraulic conductivity, and increased bulk density. In this study, soil contamination with chainsaw mineral oil increased the soil bulk density, with simultaneous deterioration of total porosity and air-filled porosity. Oil contamination can lead to anaerobic conditions in soil by smothering soil particles and blocking air diffusion into soil pores, with subsequent effects on soil microbial communities (Townsend et al. 2003; Labud et al. 2007; Sutton et al. 2013). Water resistance of soil aggregates is another property of soil that can be modified by the presence of oily substances containing aromatic hydrocarbons. In addition, crude oil-contaminated soils are generally more hydrophobic than pristine sites (Quyum et al. 2002; Aislabie et al. 2004).

The negative impact of soil contamination by petroleum products and PAHs on soil microorganisms and their metabolic activity has been confirmed by numerous experiments carried out under controlled conditions. The impact of individual PAHs and PAH mixtures on the microbiological condition of agricultural soils has been extensively investigated (Eschenbach et al. 1991; Maliszewska-Kordybach et al. 2000; Kucharski et al. 2000, 2004; Klimkowicz-Pawlas and Maliszewska-Kordybach 2003). A very important factor affecting the intensity of the influence of PAHs on soil microorganisms is the quantity and quality of soil organic matter. The bioavailability and environmental persistence of PAHs are most affected by soil organic matter content. Soil organic matter content also determines the enzyme activity and abundance of microorganisms (Veres et al. 2015; Kotroczó et al. 2014). Increased organic matter content in soil promotes stronger adsorption of PAHs, which reduces their content in the aqueous phase of the soil, and thus their bioavailability (Maliszewska-Kordybach et al. 2000). The bioavailable fraction of PAHs in soils polluted over a long period of time represents only a small percentage of their total content because of sorption by soil humic compounds (Swartz et al. 1995).

Dehydrogenases are intracellular enzymes that undergo rapid degradation after cell death, and are therefore not accumulated in the soil. Because of this property, their activity is often used in ecotoxicological research (Eschenbach et al. 1991; Maliszewska-Kordybach et al. 2000; Klimkowicz-Pawlas and Maliszewska-Kordybach 2003). The inhibition of dehydrogenases and urease detected during this experiment might be the result of direct toxic effects of PAHs on the microorganisms, but might also be an indirect result of the deterioration of soil physical properties.

Among the invertebrates, earthworms are used for testing soil environmental pollution (ISO 15799: 2003). High sensitivity of earthworms to soil contaminants, including PAHs, is because of the fact that these organisms consume harmful compounds through the digestive tract, together with soil particles. Numerous studies have confirmed the negative effects of PAHs on the survivability and reproduction of earthworms (Brown et al. 2004; Eom et al. 2007). The present study confirmed the high sensitivity of earthworms to soil contamination with mineral oil containing PAHs. Both population density and biomass of these animals were strongly decreased when oil for lubrication of chainsaws was added to the soil, and the impact of the oil on biomass and quantity of earthworms was directly proportional to the amount of oil added.

A major problem when considering the adverse effects of exposure of soil organisms to PAHs is the duration of exposure to these substances and, in the case of model experiments, the period elapsed since PAHs were administered to the soil. Studies under laboratory conditions have shown that toxicity of various PAHs changes with time. The contributing factors for this phenomenon are the intensity of sorption of hydrocarbons by soil organic matter and regulation of their bioavailability, the mechanism of adaptation of soil organisms to the presence of PAHs in the soil (Lipińska et al. 2013), as well as the possibility of interactions between PAHs and other pollutants, especially heavy metals, whose intensity varies with time (Shen et al. 2006). In this experiment, the soil samples were of one soil type, with similar soil organic matter content. Soil samples were collected from areas directly adjacent to each other, so one can assume that the impact of this factor on modification of bioavailability of PAHs contained in oil would be limited. Concentrations of PAHs measured in the experiment did not exceed the permissible levels of these compounds in the soil as per the relevant soil quality standards (Trenck et al. 1994; Jensen and Folker-Hansen 1995). In this study, the cumulative content of the five PAHs measured (phenanthrene, pyrene, chrysene, benzo(a)pyrene, and dibenz[a,h]anthracene) did not exceed 0.3 mg kg^−1^ in the soil sample where the highest amount of mineral oil was administered. However, the adverse effect of oil contamination on enzymatic activity and earthworm biomass has been established. Soil enzyme activities have been considered useful parameters to provide a biological assessment of soil quality (Andreoni et al. 2004; Alrumman et al. 2015).

The activity of microorganisms and their enzymes is also determined by the properties of hydrocarbons, such as their degree of solubility in the soil solution and the amount of benzene rings in molecules of different hydrocarbons. Hydrocarbons with two, three, and four rings (e.g., naphthalene, phenanthrene, anthracene, and pyrene) are generally susceptible to microbial decomposition, and in the early stages of experiments might cause an increase in the activity of microorganisms and their enzymes (Smreczak and Maliszewska-Kordybach 2003; Zhon et al. 2010). Hydrocarbons with more than four benzene rings are strongly adsorbed and thus poorly bioavailable. In PAH degradation experiments in soil, a biphasic process can usually be observed; a rapid decline in contaminant content will occur in the first stage, while slow decomposition takes place in the second phase. The extended contact of PAHs with soil results in the formation of strong bonds with the soil components and penetration of these compounds into the soil micropores (Semple et al. 2003; Smreczak et al. 2013). In this experiment, the enzymatic activity and population and biomass of earthworms were measured 30 days after oil had been administered to the soil. Based on the results of model tests (Maliszewska-Kordybach et al. 2000), it was assumed that after this length of time the symptoms of the negative impacts of PAHs on soil biological parameters had stabilized.

## Conclusions

Mechanization of forestry work leads to the emission of pollutants into the natural environment, including PAHs. In this study, the highest PAH concentrations were observed for chrysene and pyrene. Oil contamination modified the physical properties of forest soils. Oil at 100 g/m^2^ caused a decrease of 4 % in air-filled porosity, and the highest amount of oil (200 g/m^2^) resulted in a decrease in air-filled porosity of nearly 10 % compared with unpolluted soil. The addition of oil to soil resulted in the increased water resistance of different size aggregates; this influence was stronger the higher the amount of oil introduced into the soil. Enzyme activity was negatively correlated with PAH content. The lowest amount of oil (50 g/m^2^) resulted in a slight decrease in urease activity, while activity was reduced by 40 and 50 % at 100 and 200 g/m^2^, respectively, compared with the control. Earthworm population density reflected the state of oil-contaminated soils. The biomass of earthworms was directly proportional to the amount of oil applied.
